# Symmetry, topology and the maximum number of mutually pairwise-touching infinite cylinders: configuration classification

**DOI:** 10.1098/rsos.160729

**Published:** 2017-01-18

**Authors:** Peter V. Pikhitsa, Stanislaw Pikhitsa

**Affiliations:** Seoul National University, Seoul 151-742, Korea

**Keywords:** mutually touching cylinders, chirality matrix, topological invariant

## Abstract

We provide a complete classification of possible configurations of mutually pairwise-touching infinite cylinders in Euclidian three-dimensional space. It turns out that there is a maximum number of such cylinders possible in three dimensions independently of the shape of the cylinder cross-sections. We give the explanation of the uniqueness of the non-trivial configuration of seven equal mutually touching round infinite cylinders found earlier. Some results obtained for the chirality matrix, which is equivalent to the Seidel adjacency matrix, may be found useful for the theory of graphs.

## 1. Introduction

The problem of mutually touching infinite cylinders begets a non-trivial geometry leading to expansive or auxetic behaviour of a regular network of cylinders that may be interesting for physics [1–3]. A configuration of mutually touching *n* infinite cylinders (named an *n*-knot in [1–3] and, alternatively, *n*-cross in [3]) lies in the core of the auxetic properties because each mutual touching removes a degree of freedom from the configuration until for the configurations in the network only expansion or contraction as a whole is allowed of all possible motions [1].

In order to construct a configuration of mutually touching cylinders one may begin with two infinite round cylinders in a mutual touch at one point (like scissors) and then add the third one so that it simultaneously touches both cylinders. Then, one adds the fourth cylinder to touch simultaneously each of the other ones. Beginning from the fifth cylinder further adding the cylinders grows harder. Thus, the problem of how many infinite round cylinders in three dimensions it is possible to put into the mutual touching arises.

**Figure 1.**
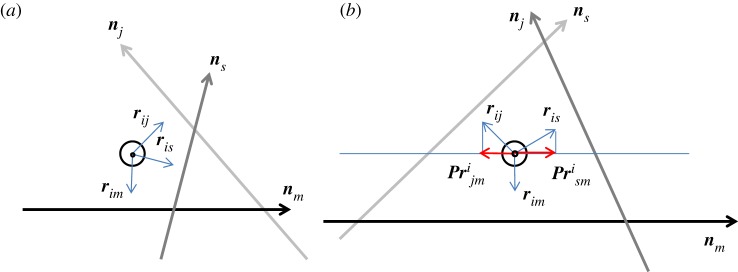
Top view of the *i*th line encaged by three lines that we call a ring. The line direction towards the reader is denoted by a circle with a dot. (*a*) No encaging; (*b*) the line encaged by the other three lines. The red arrows show the projections of the unit vectors in the direction orthogonal to ***r****_im_*.

Since the discovery of the mutually touching 7, 8 and 9 round infinite cylinders [1–4], many questions have remained open. First of all, there is the question of configuration classification, that is the development of a method that would give a possibility to distinguish configurations to find new ones. Another question is of how many cylinders of arbitrary adjustable cross-sections could mutually contact. Before only round cylinders have been under consideration [1–4]. And finally is the configuration with seven all-equal round cylinders (named here **a89** for the reasons explained below) first found in [2] (see figure 1*b* in this paper) and then rediscovered in [4] unique or are there other non-trivial configurations with seven all-equal round cylinders? We will answer these questions by considering the symmetry and topology of configurations of oriented lines in three dimensions as well as by utilizing the restrictions imposed by the conditions of mutual contacts.

We use the normalized *chirality matrix* that we introduced in [3], which describes the topology of a configuration of infinite oriented lines in three-dimensional space and reads

1.1 $$P_{i,k}\equiv \left(\begin{array}{cccc} 0 & \frac{R_{ik}[n_{i}\times n_{k}]}{\left|R_{ik}[n_{i}\times n_{k}]\right|} & \cdots & \cdots \\ \frac{R_{ik}[n_{i}\times n_{k}]}{\left|R_{ik}[n_{i}\times n_{k}]\right|} & 0 & \cdots & \vdots \\ \vdots & \vdots & \ddots & \vdots \\ \vdots & \cdots & \cdots & 0 \end{array}\right),$$

where ***n***_*i*_ is a unit vector along the *i*th line (figure 1) and ***R***_*ik*_ is the vector of the shortest distance between the *i*th and the *k*th lines, collinear with [***n***_*i*_ × ***n***_*k*_]. Note that chirality matrix *P* as a symmetrical matrix of the entries +1 and −1 and the zeroes on its diagonal is identical to the Seidel adjacency matrix used in the theory of graphs. According to its definition, the Seidel adjacency matrix of a simple undirected graph is a symmetric matrix with a row and column for each vertex, having 0 on the diagonal, −1 for positions whose rows and columns correspond to adjacent vertices, and +1 for positions corresponding to non-adjacent vertices. Therefore, some results that we obtain below for the chirality matrix may be useful for the graph theory. The chirality matrix has its inner symmetries: changing directions of lines leads to simultaneously changing the sign of the *i*th column and *i*th row and the line number permutations lead to *i*th and *j*th permutations of corresponding columns and rows. These operations are the similarity transformations that do not change the determinant of the chirality matrix, which remains the topological invariant [3].

As far as the chirality matrix *P* is not enough to tell all the nuances of the topology of configurations of mutually pairwise-touching cylinders, we introduce a novel matrix R that we name the *ring matrix*. Together with the chirality matrix *P* it can unambiguously distinguish the topology of non-trivial configurations of lines in three dimensions in general and thus can be applied for the classification of the configurations of 7, 8 and 9 mutually pairwise-touching infinite cylinders (called 7-, 8- and 9-knots in [3]). Along with R we will generate a matrix *Q* from the chirality matrix *P* that will ignore the direction of oriented lines because the orientations are irrelevant for the configurations of the cylinders. Such situation is known for nematic liquid crystals where the director rather than the vector determines their configurations.

## 2. Ring matrix and complete classification of the configurations

One can write

2.1 $$\text{and}\;\begin{array}{cc} & R_{ik}=|R_{ik}|r_{ik}\\ & r_{ik}=\frac{P_{ik}[n_{i}\times n_{k}]}{\left|P_{ik}[n_{i}\times n_{k}]\right|} \end{array}\}.$$

The unit vectors ***r****_ik_* are shown in figure 1 where the projection along the direction of the *i*th line of the configuration of four lines is given. To get the criterion of whether the *i*th line is encaged by the other three lines we project the unit vectors to define

2.2 $${Pr}_{jm}^{i}=r_{im}-(r_{im}r_{ij})r_{ij}.$$

When the projections point in different directions for each of the three lines, then the *i*th line is surrounded by the other lines as shown in figure 1*b* and thus is encaged (entangled). The criterion would be

2.3 $$\text{where}\;\begin{array}{rl} & S(\;j,s,m;\;i)+S(m,s,j;\;i)+S(\;j,m,s;\;i)\equiv -3\\ & S(\;j,s,m;\;i)=\text{sign}({Pr}_{jm}^{i}\;{Pr}_{sm}^{i}) \end{array}\}.$$

Calculated for each line it can form a vector

2.4 $${ℜ}_{i}=\underset{s>m>j}{\sum }1,\text{if}\;[S(\;j,s,m;\;i)+S(m,s,j;\;i)+S(\;j,m,s;\;i)=-3];0,\text{otherwise}.$$

Such a vector tells how many rings of three lines encage each line in a given configuration. However, we found that for the unambiguous discrimination of the topology of line configurations the vector, being a topological invariant, is still insufficient. As we mentioned earlier, the complete description stems from the *ring matrix* R. To define R, we give the vector ℜ the structure where in equation (2.4) instead of 1 s (the scalars) we sum up vectors

2.5 $$R_{i,k}=\sum_{s>m>j}\left\{ \begin{array}{ll} \left\{ \begin{array}{ll} 1, & \text{if }[(s=k)\text{ or }(j=k)\text{ or }(m=k)]\\ 0, & \text{otherwise} \end{array}\right. & \text{if }[S(\;j,s,m;\;i)+S(m,s,j;\;i)+S(\;j,m,s;\;i)=-3]\\ 0, & \text{otherwise} \end{array}\right.,$$

that indicate how many times each line enters into each of the rings encaging a given line. For illustration, we give the vector ℜ and the matrix R calculated for the configuration **a89** for seven*-knot [2,3] with the help of equations (2.4) and (2.5), respectively

2.6 $$\begin{array}{rl} ℜ(a89) & =\left(\begin{array}{c} 4\\ 8\\ 0\\ 0\\ 4\\ 4\\ 0 \end{array}\right), \end{array}$$

2.7 $$\begin{array}{rl} R(a89) & =\left(\begin{array}{ccccccc} 0 & 1 & 1 & 4 & 1 & 1 & 4\\ 4 & 0 & 4 & 4 & 4 & 4 & 4\\ 0 & 0 & 0 & 0 & 0 & 0 & 0\\ 0 & 0 & 0 & 0 & 0 & 0 & 0\\ 1 & 1 & 4 & 4 & 0 & 1 & 1\\ 1 & 1 & 1 & 1 & 4 & 0 & 4\\ 0 & 0 & 0 & 0 & 0 & 0 & 0 \end{array}\right). \end{array}$$

One can notice that the sum of the numbers in the first row in equation (2.7) is three times the first number in the vector in equation (2.6). The same is true for all the rows in equation (2.7). It is so because each ring encaging a line consists of three other lines. The matrix R is a topological invariant which together with the chirality matrix [3]

2.8 $$P(a89)=\left(\begin{array}{ccccccc} 0 & +1 & +1 & +1 & +1 & +1 & +1\\ +1 & 0 & +1 & +1 & +1 & -1 & +1\\ +1 & +1 & 0 & -1 & -1 & -1 & +1\\ +1 & +1 & -1 & 0 & -1 & +1 & +1\\ +1 & +1 & -1 & -1 & 0 & +1 & -1\\ +1 & -1 & -1 & +1 & +1 & 0 & +1\\ +1 & +1 & +1 & +1 & -1 & +1 & 0 \end{array}\right),$$

completely and unambiguously describes the topology of the line configuration. It is remarkable that one can reproduce the chirality matrix and the ring matrix just by inspecting any given configuration while establishing directions along the cylinders and marking the chirality at each contact, along with counting the number of rings around each cylinder that contain this cylinder. The reverse problem can be solved as well: being given both matrices one can reproduce the topologically equivalent line configuration. However, for practical purposes of finding new configurations instead of inspecting the two matrices it would be easier to have a numerical invariant that could distinguish different topologies.

## 3. A numerical invariant for the complete classification of configurations

Unfortunately, the chirality matrix is sensitive to the opposite orientations of the lines: if the direction of a line is switched to the opposite one then the corresponding matrix entries are multiplied by −1 and the chirality matrix becomes different. On the other hand, any numerical invariant based on the chirality matrix should not depend on switching line direction to opposite (because cylinders are symmetric) and thus one has to construct a matrix that is degenerate towards opposite line directions. Therefore, we introduce a new matrix *Q* derived from *P* which is invariant with respect to the line orientation as follows (we are using equation (2.8) as an example):

One has to transform the first row in *P*(*a*89) into all +1 s (it is already so) and to sum up numbers in the corresponding columns and put the sums into the first row.
One has to transform the second row in *P*(*a*89) into all +1 s (here by reverting sign in the sixth column and then in the sixth row) then to sum up numbers in the corresponding columns and put the sums into the second row.

After proceeding in the same way through the whole matrix of equation (2.8) one gets the symmetric matrix

3.1 $$Q(P(a89))=\left(\begin{array}{ccccccc} 6 & 4 & 0 & 2 & 0 & 2 & 4\\ 4 & 6 & 2 & 0 & -2 & -2 & 2\\ 0 & 2 & 6 & -2 & 0 & 2 & 2\\ 2 & 0 & -2 & 6 & -2 & 2 & 4\\ 0 & -2 & 0 & -2 & 6 & 0 & 0\\ 2 & -2 & 2 & 2 & 0 & 6 & 0\\ 4 & 2 & 2 & 4 & 0 & 0 & 6 \end{array}\right).$$

An additional advantage of *Q* is that it is different for the mirror configuration (which means that configuration *a*89 has been reflected in the mirror)

3.2 $$Q(-P(a89))=\left(\begin{array}{ccccccc} 6 & -2 & 2 & 0 & 2 & 0 & -2\\ -2 & 6 & 0 & 2 & 4 & 4 & 0\\ 2 & 0 & 6 & 4 & 2 & 0 & 0\\ 0 & 2 & 4 & 6 & 4 & 0 & -2\\ 2 & 4 & 2 & 4 & 6 & 2 & 2\\ 0 & 4 & 0 & 0 & 2 & 6 & 2\\ -2 & 0 & 0 & -2 & 2 & 2 & 6 \end{array}\right).$$

Equations (3.1) and (3.2) satisfy the equation that holds for all seven-knots

3.3 $$Q(P)+Q(-P)=\left(\begin{array}{ccccccc} 12 & 2 & 2 & 2 & 2 & 2 & 2\\ 2 & 12 & 2 & 2 & 2 & 2 & 2\\ 2 & 2 & 12 & 2 & 2 & 2 & 2\\ 2 & 2 & 2 & 12 & 2 & 2 & 2\\ 2 & 2 & 2 & 2 & 12 & 2 & 2\\ 2 & 2 & 2 & 2 & 2 & 12 & 2\\ 2 & 2 & 2 & 2 & 2 & 2 & 12 \end{array}\right).$$

The form of equation (3.3) also holds for all eight- and nine-knots where the matrices are 8 × 8 and 9 × 9 and the numbers on the diagonal of the corresponding matrix are 14 and 16, respectively.

All possible numerical invariants could be formed from the traces of corresponding matrices such as: $\text{tr(}R^{2}\text{) }$, $\text{tr(}R^{3}\text{) }$, … $\text{tr(}R^{7}\text{) }$ (see, for example, p. 270 in [5]). Yet, in order to distinguish mirror configurations we have to also include *Q*, for example, by adding $\text{tr(}QR\text{) , }\;\text{tr}(QR^{2}),\;\text{tr}(Q^{2}R)$, etc. Such numbers could be combined in a single numerical for each configuration. Here, we suggest a simple formal summation over *n* = 0,1,2… that proved to be a good practical numerical invariant that could tell the topologically different configurations

3.4 $$℘(P,R)=\underset{n}{\sum }\text{tr(}QR^{n}\text{) }=\text{tr}[Q{\left(1-R\right)}^{-1}].$$

One can introduce an additional ring invariant that would have the same value for the configurations with the same ring matrix, which is insensitive to mirroring

3.5 $${℘}_{R}=℘(P,R)+℘(-P,R).$$

It directly follows from equation (3.4) along with the equation (3.3) that equation (3.5) is determined only by R.

Below we will give the results of the calculations of invariants for a number of configurations.

## 4. The chirality matrices forbidden for mutual pairwise touching

We will show that much information about *n*-knot configurations can be extracted solely from the analysis of the chirality matrix. The most important is that there is a rigorous sufficiency criterion as to chirality matrices that lead to impossible configurations, that is to those configurations that never allow mutual pairwise contacts of all cylinders. Namely,

– *if P contains a subset of five cylinders that have a* 5 × 5 *submatrix (which we conventionally call K*5 *from the theory of graphs* [6])
  4.1 $$K5=\left(\begin{array}{ccccc} 0 & 1 & 1 & 1 & 1\\ 1 & 0 & 1 & 1 & 1\\ 1 & 1 & 0 & 1 & 1\\ 1 & 1 & 1 & 0 & 1\\ 1 & 1 & 1 & 1 & 0 \end{array}\right),$$
  *or any of its transformation with changing directions and cylinder permutations (simultaneous changing the sign of ith column and ith row or/and ith and jth permutations), as well as mirror transformation that changes the signs of all entries of K5, then this configuration is impossible for mutual pairwise contacts*.

**Figure 2.**
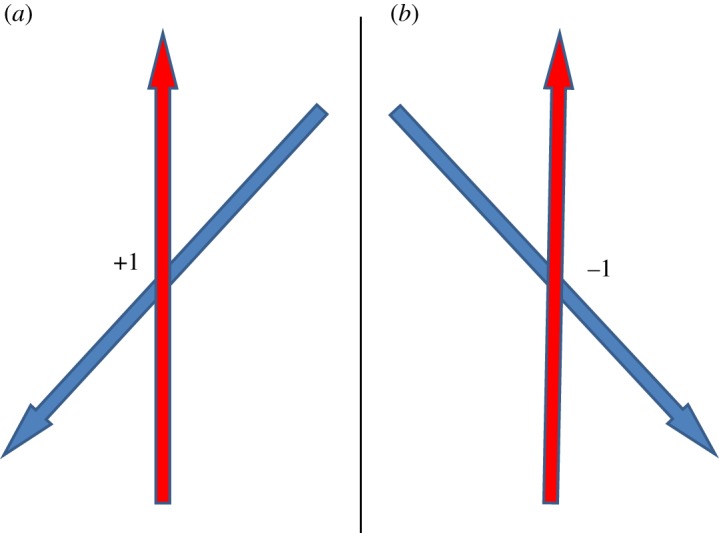
The elementary diagrams illustrating the chirality +1 (*a*) and −1 (*b*) of the configuration of two oriented lines in three dimensions.

To prove the impossibility of *K*5 to have all the cylinders in mutual contact we will use a projection diagram to form the matrix *K*5. First, one can draw a diagram element to illustrate the assignment of signs +1 and −1 for the projection of two cylinders (figure 2): nearest rotation from red to blue going counter-clockwise gives +1 for the corresponding element of the chirality matrix, otherwise −1. Here, in the diagram the red line should run over the blue line.

**Figure 3.**
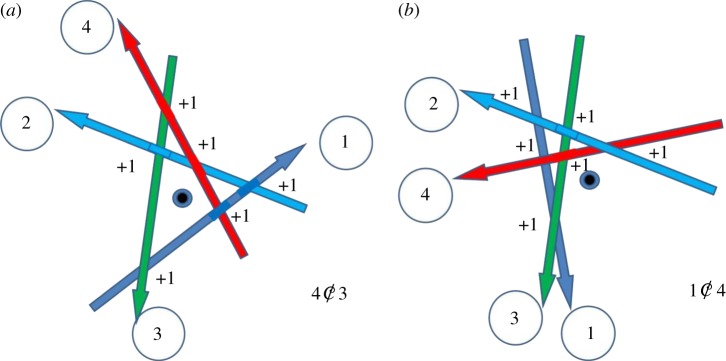
(*a*) Illustration of *K*5 where cylinder 4 does not contact cylinder 3 marked with the crossed letter C. Zeroth cylinder points out of the picture. (*b*) The other case where first cylinder does not contact with the 4th cylinder.

It is not difficult to construct a diagram for *K*5 and prove the theorem by direct inspection of the configuration, which we give below in figure 3. The determinant of *K*5 is either 4 or −4 (for the mirror configuration) and only *K*5 can have such determinants. We proved it by trying all possible 5 × 5 symmetrical matrices with the null diagonal. This property can be used to find the presence of *K*5 in any chirality matrix. The requirement that *K*5 should be absent selects out of all possible chirality matrices *P* for 7 × 7 matrices with all possible determinants which absolute values are

4.2 2,6,10,14,18,22,26,30,34,42,46,50,54,66,70,78,90,102,150,162,250,

only the matrices of determinants with absolute values

4.3 2,10,18,42,54,66,102,150,162,250,

which do not contain *K*5. We found existing configurations for all determinants of equation (4.3) (figure 7 and electronic supplementary material, appendices 1,3,4) but for 250.

There may exist configurations, which do not contain *K*5 but still cannot have all the cylinders in mutual contact. Namely, of all the matrices of equation (4.3) only for |*P*| = 250 all cylinders cannot be in mutual contact. Below, we show that *P*250 in spite of not having *K*5 still cannot be realized with all mutual contacts for positive radii of the cylinders: at least two of the cylinders cannot contact.

Let us introduce a useful definition. Two cylinders are said to be in *equal environment* (EE) if two rows/columns that correspond to these two cylinders in the chirality matrix are identical or can be made identical by multiplying by −1. Of course, one should ignore the diagonal zeroes. The necessary condition for a matrix to have such a property is to have 1 or −1 among its eigenvalues. Yet, some chirality matrices (for example, the one with |*P*| = 42) while having 1 and −1 among its eigenvalues do not have EE. The EE property is important for understanding why *P*250 configuration is impossible for mutual contacts. Later, we will show that EE also helps to select out those configurations that cannot have all equal cylinders.

**Figure 4.**
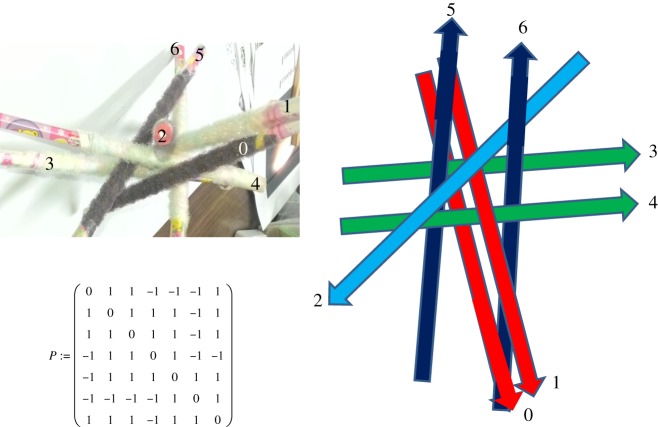
A pencil model of the configuration of cylinders *P*250 (left) and its schematic presentation with directed lines. The numbers of lines correspond to the numbers on the pencils.

From the matrix *P*250 shown in figure 4, one can see that the first and the second cylinder are in EE. All other chiral matrices out of the list of equation (4.3), for example, for the configuration **b14** with the chiral matrix determinant −102 containing cylinders in EE, possess a property:

*If one switches the chirality between the very two EE cylinders in the matrix, then the matrix turns into the matrix which contains K5*.

The only exception is the chiral matrix *P*250: if one switches the chirality matrix entry between the first and the second cylinders in EE (which is *P*_1,2_ = 1 in figure 4) to *P*_1,2_ = −1, then the determinant will switch to −250 and will not contain *K*5, unlike other chiral matrices of equation (4.3) that have EE. This specific property greatly restricts the possible configurations of the cylinders corresponding to *P*250. Indeed, the two cylinders in EE cannot go through any ring similar to the one in figure 3*a* which is created by the lines 1, 2, 3. We call such a ring *right wreath*. In this picture, the lines 0 and 4 are in EE and both punch the ring. The configuration is *K*5, yet if we switch the chirality between lines 0 and 4 then there is no *K*5. That means that if the two EE cylinders in a configuration with |*P*| = 250 punched any of such rings, then after switching their chirality, *K*5 would appear as it happens for other chiral matrices. On the other hand, if the two EE cylinders are located outside the ring made by lines 1, 2, 3 in figure 3*b* (we call such a ring *right scissors*) then again they would be in *K*5 configuration. Switching the chirality between the two removes *K*5 which never happens for *P*250. The restrictions of not having such rings to punch and to pass by, reduce the configuration for *P*250 down to the one shown in figure 4. The schematic with lines in figure 4 demonstrates that the second cylinder is in the see-saw configuration balancing on the first cylinder and is being able to touch either the third or the fourth cylinder but never both.

Let us note that while investigating the matrix *P*250, we serendipitously came across a remarkable property of the matrix: we found that the determinant |*P_i,k_*(*r_i_* + *r_k_*)| ≥ 0 for all *r_i_* ≥ 0. We proved it analytically by direct calculation finding the series of all positive terms for the determinant. A similar determinant with all *P_i,k_* = 1 was considered long ago in [7].

Finally, note an interesting parallel between the famous Kuratowski's theorem [6] that if a generic graph is non-planar then it should contain as a subgraph either *K5* or *K3,3*, and our finding that the cylinders cannot be all in mutual touch if their chirality matrix contains either *K5* or *P250*.

## 5. The maximum number of mutually pairwise-touching straight cylinders of arbitrary cross-section

The question of the number of cylinders of arbitrary cross-section arises when one tries to use the arguments of the degrees of freedom that we used for 7, 8 and 9 round cylinders [1–3]. Solely on the arguments, it would be reasonable to expect that while arbitrary cross-sections have an infinitely large number of shapes and therefore degrees of freedom, then the number of mutually pairwise-touching straight cylinders of arbitrary cross-section could be infinite. However, the theorems given below claim that this is not the case: the number of mutually pairwise-touching straight cylinders is restricted from above at least by the number 14.

The notion of ‘pairwise’ means here that the point of a contact is shared by two and only two cylinders. The degree of freedom arguments used in our previous works [1–3] give a definite prediction as to the maximum possible number of cylinders of arbitrary radii (which is 7) provided that the radii are not adjusted. If the radii are involved in calculations, then the maximum number increases up to nine because each adjustable radius adds a degree of freedom. However, there is a question: what if the cross-section of cylinders is not a circle but is possible to be varied and adjusted? It is easy to imagine an elliptical cross-section, which immediately produces up to two additional degrees of freedom for each cylinder (rotation around the cylinder axis and the aspect ratio). It can be generalized for infinitely many degrees of freedom for arbitrary shaped cross-sections. As we said earlier, one would expect an arbitrary number of mutually touching cylinders. Yet, with the help of the chirality matrix (using the fact that any (*n* + 1)-dimensional chirality matrix contains *n* submatrices being *n*-dimensional chirality matrices) we will show that it is not the case for pairwise touching, namely:

the maximum number of mutually pairwise-touching infinitely long straight cylinders with arbitrary adjustable cross-sections is restricted from above.

The degeneracy of a straight line when several lines pass through one single point in space is not considered because in such a degenerate case the notion of ‘mutual contact’ does not have a meaning of a pairwise contact which we explore. Still it is possible to consider infinite straight stripes (blades) with straight line edges if only the pairwise contact is allowed, which we will publish elsewhere.

*P*250 plays the role of *K*5 for larger than seven number of cylinders. The presence of *K*5 or *P*250 is a sufficient condition for any configuration of cylinders of arbitrary cross-section not to have all the cylinders in mutual pairwise contacts. We use the properties of *K*5 and *P*250 to prove important theorems:

1. The number of arbitrary cylinders that can be in mutual pairwise contacts cannot exceed 18. That means that starting from *n* > 18 any chirality matrix contains *K*5.
2. Moreover, the number of arbitrary cylinders in mutual pairwise contacts cannot exceed 14. This happens because starting from *n* > 14 any chirality matrix contains *P*250 as a submatrix.

We prove these theorems by direct calculations (the text of the program for finding *K*5 is given in electronic supplementary material, appendix 5) showing that:

*All chiral matrices larger than 18 × 18 contain K5. There is only one prototype matrix 18 × 18 (up to the similarity transformations) that does not contain K5*:

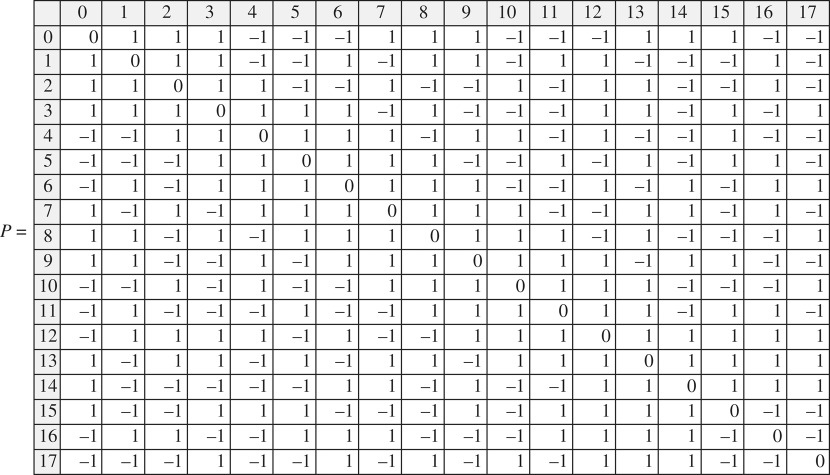


This matrix has a simple characteristic polynomial (*x*^2^ − 17)^9^.

*All chirality matrices larger than 14* × *14 contain P*250*. There is only one prototype matrix 14* × *14 (up to the similarity transformations) that does not contain both K*5 *and* 250:


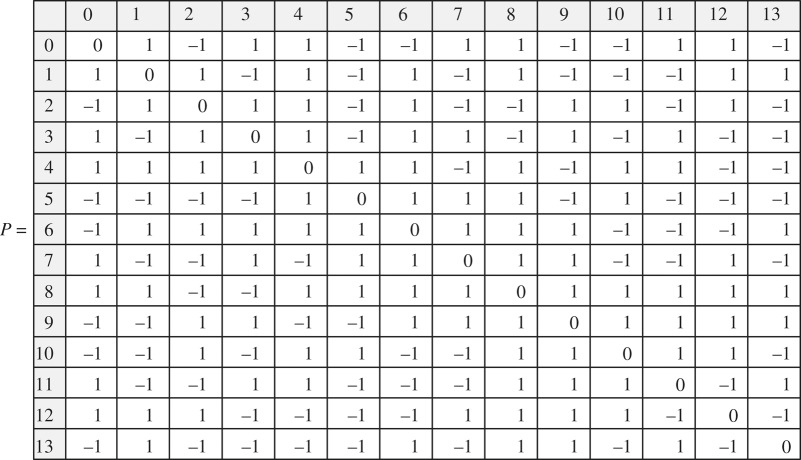


This matrix has a simple characteristic polynomial (*x*^2^ − 13)^7^.

The direct calculation was performed by using a MathCad11 solver programmed to find *K*5 and *P*250 matrices in all possible chirality matrices of successively growing size. Briefly, the code algorithm uses recursive/iterative exhaustive search and is as follows. First, we look up through all possible 5 × 5 symmetric matrices with the zero diagonal and all possible combinations of entries ±1. Keep those matrices that have a ‘unique’ set of eigenvalues, which means that if during sorting one comes across a matrix with the same set of eigenvalues as the set of a previously found matrix of the unique set, such a matrix is ignored as not being unique. Those matrices that have the set of eigenvalues, which coincides with the set of *K*5 are also ignored. Second, we add an additional row (along with the transposed column to keep the matrix symmetric) with one zero and all other possible entries ±1 to each of the unique matrices of the first step to obtain a set of 6 × 6 symmetric matrices. While trying all possible entries ±1 for the attached row/column for each of the matrices we select a ‘unique’ set of 6 × 6 symmetric matrices. Then of the selected ‘unique’ ones, we sort out those that have 5 × 5 symmetric submatrices with the same set of eigenvalues as *K*5 has. Third, the procedure of adding a row/column to the unique matrices and the sorting are repeated again and again until there is no matrix left in the set. As is seen, the largest matrix that can be obtained in this way is the 18 × 18 matrix given above. The text of the program for finding *K*5 is given in electronic supplementary material, appendix 5. A complete calculation up to the matrix 18 × 18 may take up to 4 hours depending on the CPU.

## 6. Collection of configurations of seven, eight and nine mutually touching round infinite cylinders

**Figure 5.**
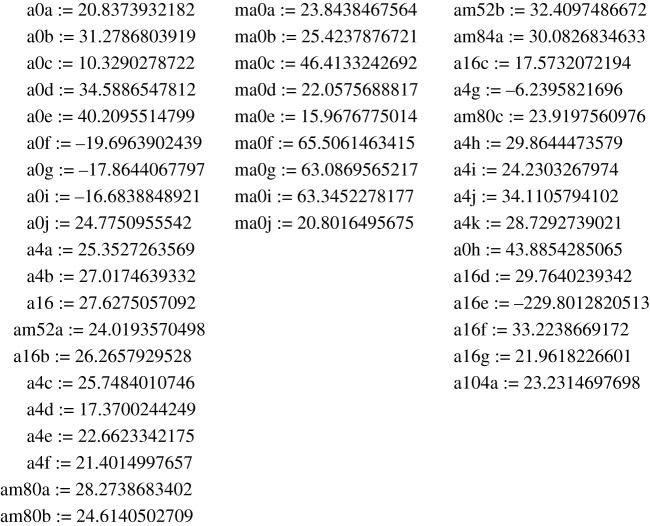
The invariants of all nine-knot configurations found.

In order to analyse and classify different configurations, we produced a collection of seven-knots (see figure 8 below and electronic supplementary material, appendices 1,3,4). For this purpose, we first calculated several nine-knots (in addition to the one first published in [3] with the determinant of the chirality matrix (see equation (1.1) for the definition) |*P*| = 0, which we designated first as **a** and then as **a0a** (when the number of configurations found grew big) with determinants −52, 16, 4, 0 which we first designated **b**, **c**, **d** and **e** when they were few and then changed to **am52a**, **a16**, **a4a** and **a0b**, correspondingly, and others (figure 5 and electronic supplementary material, appendix 2 for the complete list). Here, **a = a0a** is different from **e = a0b** in spite of having the same zero determinant of *P*. Then, we extracted a number of seven-knot configurations by removing any two cylinders, designating the resulting configuration as **a89**, for example, when it was extracted from **a** by removing the eighth and ninth cylinders or say **e69** if it were the sixth and ninth cylinders from **e**. At the same time we produced eight-knots in the same manner and designated them accordingly (figure 8), for example **a8** when the eighth cylinder was removed from **a = a0a**. Except those configurations that originate from different nine-knots we produced a number of configurations of nine-knots and eight-knots independently. They have their own labels usually containing the information of the determinant of the chirality matrix of a certain configuration. The letter **m** before the name of a configuration means a **mirror** configuration of the one without the letter.

Many topologically different seven-knot configurations have the same determinant |*P*|, positive or negative, which had the form 4*n* + 2, where *n* is an integer. The chirality matrix turned out to be different for many configurations with the same determinant but for all cases except *n* = 0,−1, the matrices could be transformed into each other by permutations and/or by changing the sign of the row/column which corresponds to permutations between the cylinders or to a change of the direction of the orientation of a given cylinder which transformation does not change the topology of the configuration. The case *n* = 0,−1 was slightly different because the chirality matrices could have a different characteristic polynomial while having the same determinant. As we said before, the chirality matrix and its determinant could distinguish only configurations that belong to different classes with different determinants. In our paper [3], we distinguished by this means two configurations (now we identify one of them as **e69**) with |*P*| = 10, first of any seven-knot configurations discovered in 2004 [1] and **a89** (it was denoted as seven*-knot in [3]) with equal radii with |*P*| = −18, discovered in 2009 [2,3] and rediscovered in [4]. Note that for the eight-knot and nine-knot the determinants take values 4*n* + 1 and 4*n*, respectively.

For nine-cross, we obtained several cases with determinants with absolute values 0, 4, 16, 52, 80, 84, 104 (encrypted in the names of configurations) which invariants are given in figure 5.

The first configuration ever found **a = a0a** was given in [3] (electronic supplementary material, appendix 2).

**Figure 6.**
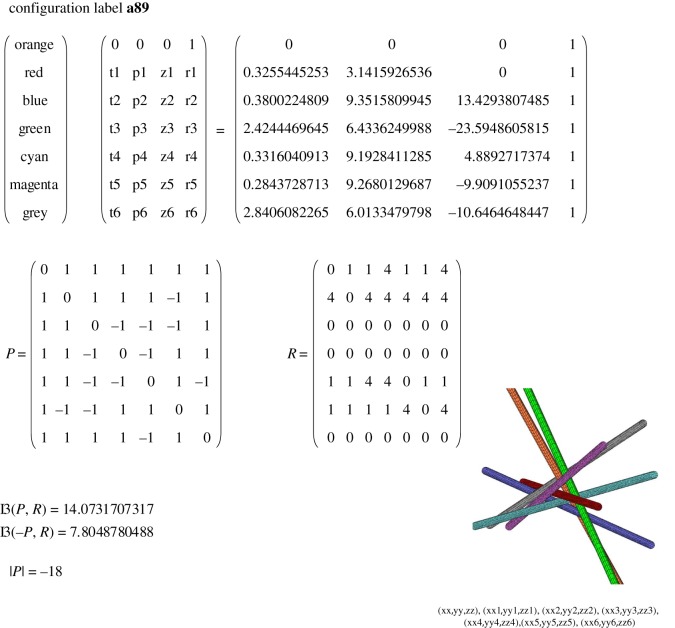
The characteristics of **a89** configuration. The numerical invariant ℘ is designated here as I3(*P*,*R*).

**Figure 7.**
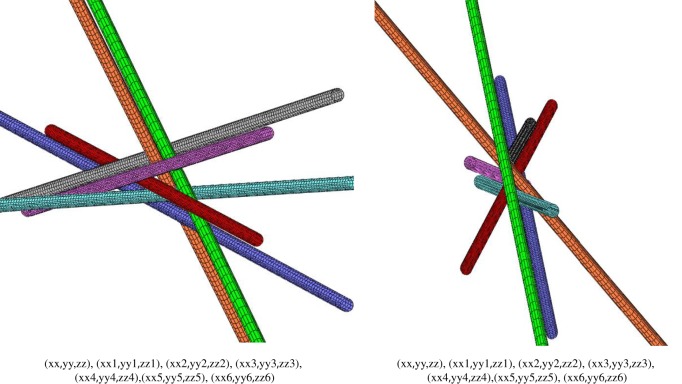
Projections display a beautiful property of any seven-knot configuration that three unit vectors orthogonal to the directions of corresponding pairs of the cylinders be nearly coplanar. Quantitatively, the enclosed volume as the coplanar check for the three unit vectors is less than circa 0.01 for the arbitrary scissors angle *t*1. At a certain scissors angle this value can be exactly zero. Both projections are shown on the example of one and the same **a89** configuration.

**Figure 8.**
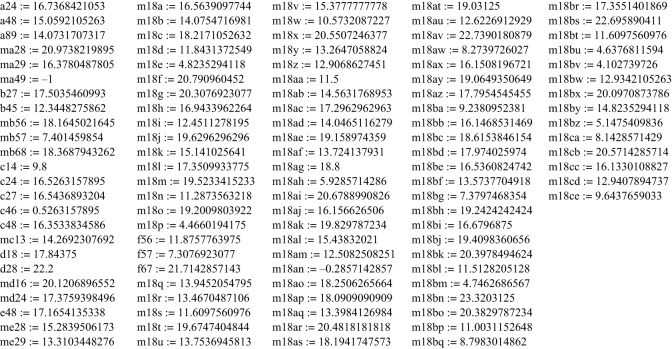
The list of different invariants of seven-knot configurations with the chirality matrix determinant −18.

For the seven*-knot or **a89**, we obtain ℘=14.(07317) and ℘=7.(80487) for the mirror configuration **ma89**. In figure 8, we give ℘ for all seven-knots with |*P*| = −18, extracted from the known nine-knots (for other determinants see electronic supplementary material, appendices 1,3,4 for the parameters of each configuration). We give in figure 6 as an example of one of the tables from electronic supplementary material, appendix 4 the unique configuration **a89**. The same configuration is shown in figure 7 in two different projections to emphasize a beautiful property of any of the seven-knots to have projections where cylinders look parallel in pairs. In figure 6, the matrix has the entries *t*1, *t*2,…, *t*6 and *p*1, *p*2, …, *p*6 which are latitude and longitude angles of the spherical coordinates of the oriented axes of the cylinders with corresponding numbers, the zero cylinder is the vertical pivot cylinder in this coordinate system that lies along the axis *z*. This coordinate system is identical to the one used in [3]. The heights of the touching points of the cylinders with the pivot zeroth cylinder are *z*1, *z*2, …, *z*6. The radii of the cylinders are *r*1, *r*2, … *r*6 correspondingly, and the radius of the zeroth cylinder is always 1.

We put in figure 8 only non-equivalent configurations with different invariants. Equivalent configurations like **a89** and **b47** should be the same as far as they have the identical ℘=14.(07317) so we omitted **b47** and the like from the list. A direct inspection of the image of the configurations confirms that they are topologically identical. The same is true for other coincidences.

**Figure 9.**
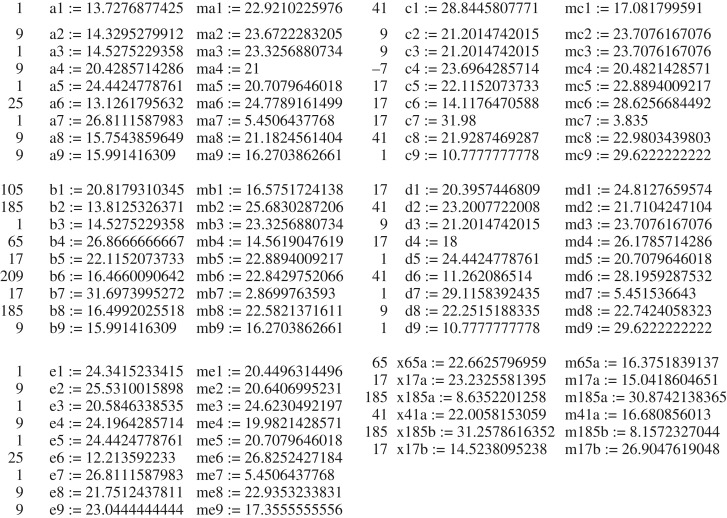
The invariants ℘ for eight-knots. Before the name of each configuration, we give the determinant of the corresponding chirality matrix. Note that in case of the even chirality matrix its determinant is the same for the mirror configuration. Stray eight-knot configurations obtained in various ways not from any of nine-knots are marked with the letter x and also given with their mirror ones. For reader's attention, we put some of the names of topologically equal configurations: **a3 = b3**; **a9 = b9**; **c2 = c3 = d3**; **b5 = c5**; **a5 = d5 = e5**; **c9 = d9**; **a7 = e7**.

Using the number invariant of equation (3.4) gives a powerful tool of complete classification and control over topologically different configurations. We can illustrate the selectivity properties of the number invariant of equation (3.5) on the example of **e9** and **d9** configurations of eight-knots that have different invariants and even have different determinants |*P*| = 9 and |*P*| = 1, respectively, as one can see from figure 9. Still ${℘}_{R}(\text{e}9)={℘}_{R}(d9)=40.4$ and if the numerical invariant works well it should indicate that both configurations have the equal ring matrices. We make sure that they indeed have one and the same ring matrix

6.1 $$R(e9)=R(d9)=\left(\begin{array}{cccccccc} 0 & 3 & 3 & 7 & 5 & 3 & 7 & 5\\ 5 & 0 & 7 & 5 & 7 & 5 & 5 & 5\\ 0 & 0 & 0 & 0 & 0 & 0 & 0 & 0\\ 0 & 0 & 0 & 0 & 0 & 0 & 0 & 0\\ 1 & 1 & 5 & 5 & 0 & 1 & 1 & 1\\ 0 & 0 & 0 & 0 & 0 & 0 & 0 & 0\\ 0 & 0 & 0 & 0 & 0 & 0 & 0 & 0\\ 2 & 2 & 4 & 2 & 2 & 8 & 4 & 0 \end{array}\right).$$

The ring matrix gives the information as to how many of the cylinders are not knotted, which is the number of rows with all zeroes. There are configurations of seven-knots that have only two unknotted cylinders (electronic supplementary material, appendix 4) with configurations: **m18r**, **m18ab**, **m18aq**, **m18cc**, **x42aa**, **x42aj**, **m102 k**, **m150n**, **m150o**, **m150q** and **m102 g**.

## 7. Configurations of equal radii for the seven-knot

Surprisingly, the chiral matrix alone can tell whether a configuration can have equal radii for all cylinders or not. It is easy to understand that the configuration with EE cannot have all cylinders being of equal radius because even the two cylinders being in EE cannot have equal radii. Suppose they have. The scissors angle between them can be made arbitrarily small because no other cylinder hinders their mutual rotation at least in one direction as far as they are in EE. But then all other cylinders are forced to lie on these two cylinders in two planes from both sides. In one of the planes, there are at least three cylinders. One of them lying in the plane and being between the other two inevitably blocks the other two from the mutual contact. Not having EE is the necessary condition for the configuration to have all equal cylinders.

The configurations with the chirality matrices that do not have EE can have equal radii of the cylinders but only asymptotically, except **a89**. As we discuss below, this very configuration does not have a possibility for *parallel* cylinders of equal radii to exist because of the topological impossibility of the Necker cube configuration. A collection of equal radii configurations is given in electronic supplementary material, appendix 3. They are not many and some of them are non-trivial though contain a pair of parallel cylinders: those are **ma37** and **ma49**. Interestingly, when the radii of the cylinders in both configurations approach 1 the large triangle extends self-similarly with the sharp angle of approximately 15° for **ma37** and 22° for **ma49**.

## 8. The uniqueness of the non-trivial configuration of seven mutually touching round infinite cylinders of equal radii

**Figure 10.**
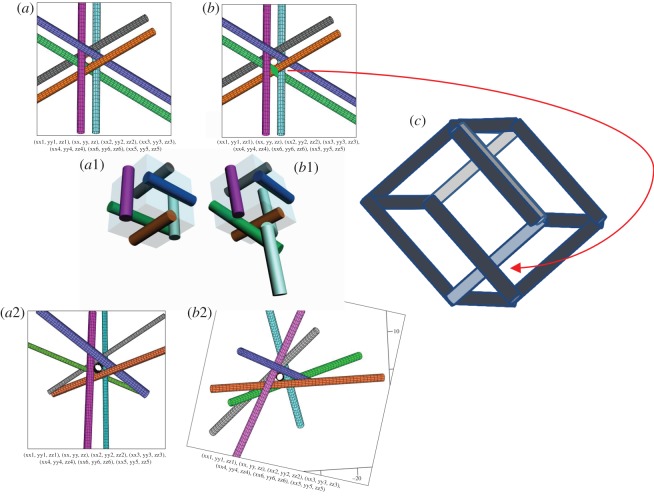
The ‘cube’ and the Necker cube.

The uniqueness of configuration **a89** of seven mutually touching round infinite cylinders of equal radii has been suspected after the publication of [4] that independently rediscovered in 2013 the same configuration that we reported in 2009 [2]. Now we will produce the arguments that support its uniqueness. Any other configuration with equal radii should approach a degenerate one with some cylinders nearly parallel that we call ‘cube’ (figure 10*a* and electronic supplementary material, appendix 3). Many configurations, such as **c24**, **md24**, **m18c**, etc. with |*P*| = −18 are such.

The specific topology of configuration **a89** makes it similar to an impossible cube (the Necker cube [8]) given in figure 10*c*. Let us show this with the help of the plates in figure 10. If one compares figure 10*a*,*b* one finds that the only difference is that the green cylinder goes over the brown one in figure 10*b*. Figure 10*b*1 shows that it is impossible for the parallel cylinders unless there is a break in the cylinder. The red arrow connector shows the similarity to the Necker cube in figure 10*c*. The plates figure 10*a*2,*b*2 show the real configurations of mutually touching cylinders topologically equivalent to figure 10*a*,*b*, respectively. Again, the only difference in both configurations is the switching between the green and the brown cylinders.

**Figure 11.**
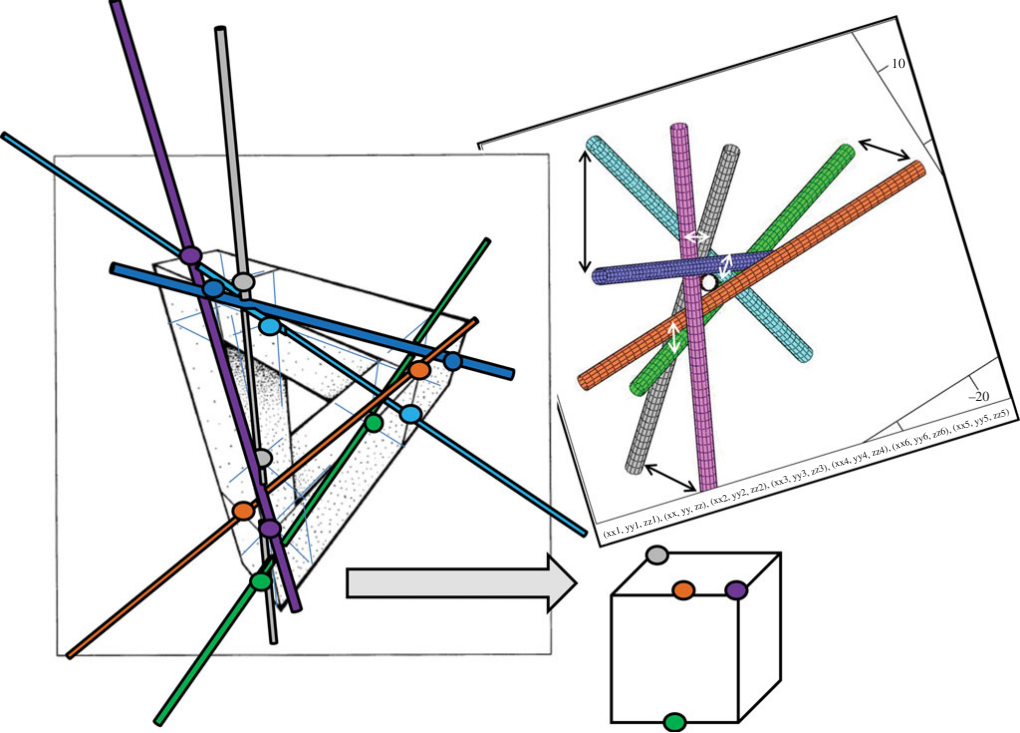
Schematic of **a89** (given in the right plate) on the background of the impossible Penrose triangle (left).

The unique topology of **a89** is revealed even more if one notices that it fits the impossible Penrose triangle [9,10] (figure 11). Indeed, one can uniformly decorate the three ‘corner’ cubes of the impossible Penrose triangle with balls as it is shown in figure 11. After coloration, the balls on the corner cubes determine the position of the lines of the corresponding colours that pass through them. The configuration a89 is reproduced when three pairs of lines (brown–green, cyan–blue and violet–grey) in figure 11 are aligned along the three sides of the triangle so that while going around the central hole (which should be filled with the red pivot cylinder to complete **a89**) and starting from the brown–green pair one can see the right-hand screw of the pair, then the left-hand screw, and finally, the left-hand screw of the violet–grey pair of lines.

On the right plate, the arrows show the pairs of cylinders in a top view of the pivot cylinder of **a89** so that the white arrows mark the parts of one pair that are squeezed between the ‘legs’ of the other pair marked with the black arrows. Configuration **a89** has its mirror **ma89** and no other configurations with non-parallel all equal cylinders are possible. The configuration **m18c** that can have asymptotically equal cylinders, given in electronic supplementary material, appendix 3, is the closest to **a89** geometrically because it has a similar chirality matrix and a similar ring matrix. However, as one can see, its invariant is different and its image is identical to the ‘cube’ of figure 10*a* with parallel cylinders.

## 9. Conclusion

We suggested a classification scheme for mutually pairwise-touching infinite cylinders. Our approach relies on the properties of the chirality matrix and the newly introduced ring matrix. The chirality matrix contains enough information to restrict possible cylinder configurations and establish the upper limit for the number of mutually pairwise-touching infinite cylinders of adjustable cross-sections. The chirality matrix helps to point out those configurations that may have equal radii in the case of round cylinders. The Necker cube topology of the non-trivial equal radii configuration of the seven cylinders found earlier stipulates its uniqueness. Finally, revealing the topology characteristics of cylinder configurations presented here may help to understand and quantify the difference and similarities in auxetic behaviour between our approach of the touching cylinder regular network [1–3] and the auxetic geometry of expanding periodic bar-and-joint frameworks recently published in [11].

## Supplementary Material

Supplementary docx file contains a collection of possible configurations of mutually touching 7 and 9 cylinders together with their topological characteristics in Appendices 1-4. Appendix 5 gives the text of a Mathcad program that calculates the chirality matrices of different dimensions that do not contain K5.

## Data accessibility

The datasets supporting this article have been uploaded as part of the electronic supplementary material.
