# Supplementary material for: Symmetry, topology and the maximum number of mutually pairwise-touching infinite cylinders: configuration classification
Source: R Soc Open Sci. 2017 Jan 18;4(1):160729. doi: 10.1098/rsos.160729 (PMC5319341; doi:10.1098/rsos.160729)
Supplement: Supplementary docx file contains a collection of possible configurations of mutually touching 7 and 9 cylinders together with their topological characteristics in Appendices 1-4. Appendix 5 gives the text of a Mathcad program that calculates the chirality matrices of different dimensions that do not [file rsos160729supp1.docx]

**Supplementary Material**

Appendix 1

7-cross invariants

Appendix 2

9-cross invariants

Appendix 2

9-cross

Configuration label a104a

Appendix 2

9-cross

Configuration label a0j

Appendix 2

9-cross

Configuration label a16g

Appendix 2

9-cross

Configuration label a16f

Appendix 2

9-cross

Configuration label a0i

Appendix 2

9-cross

Configuration label a16e

Appendix 2

9-cross

Configuration label a16d

Appendix 2

9-cross

Configuration label a0h

Appendix 2

9-cross

Configuration label a4k

Appendix 2

9-cross

Configuration label a0g

Appendix 2

9-cross

Configuration label a0f

Appendix 2

9-cross

Configuration label a4j

Appendix 2

9-cross

Configuration label a0e

Appendix 2

9-cross

Configuration label a4i

Appendix 2

9-cross

Configuration label a4h

Appendix 2

9-cross

Configuration label am80c

Appendix 2

9-cross

Configuration label a4g

Appendix 2

9-cross

Configuration label a16c

Appendix 2

9-cross

Configuration label am84a

Appendix 2

9-cross

Configuration label am52b

Appendix 2

9-cross

Configuration label am80b

Appendix 2

9-cross

Configuration label am80a

Appendix 2

9-cross

Configuration label a4f

Appendix 2

9-cross

Configuration label a4e

Appendix 2

9-cross

Configuration label a4d

Appendix 2

9-cross

Configuration label a0d

Appendix 2

9-cross

Configuration label am52a

Appendix 2

9-cross

Configuration label a16a

Appendix 2

9-cross

Configuration label a4b

Appendix 2

9-cross

Configuration label a4a

Appendix 2

9-cross

Configuration label a0c

Appendix 2

9-cross

Configuration label a0b

Appendix 2

9-cross

Configuration label a0a

Appendix 2

9-cross

Configuration label a16b

Appendix 2

9-cross

Configuration label a4c

Appendix 3

Configurations with asymptotically equal radii.

7-cross

Configuration label **a59**

orange

red

blue

green

cyan

magenta

gray

Appendix 3

7-cross

Configuration label **m18c**

orange

red

blue

green

cyan

magenta

gray

Appendix 3

7-cross

Configuration label **ma49**

orange

red

blue

green

cyan

magenta

gray

Appendix 3

7-cross

Configuration label **ma37**

orange

red

blue

green

cyan

magenta

gray

Appendix 4

7-cross

Configuration label m162j

Appendix 4

7-cross

Configuration label m162i

Appendix 4

7-cross

Configuration label m162h

Appendix 4

7-cross

Configuration label m162g

Appendix 4

7-cross

Configuration label m162f

Appendix 4

7-cross

Configuration label m162e

Appendix 4

7-cross

Configuration label m162d

Appendix 4

7-cross

Configuration label m162c

Appendix 4

7-cross

Configuration label m162b

Appendix 4

7-cross

Configuration label m162a

Appendix 4

7-cross

Configuration label m150q

Appendix 4

7-cross

Configuration label m150p

Appendix 4

7-cross

Configuration label m150o

Appendix 4

7-cross

Configuration label m150n

Appendix 4

7-cross

Configuration label m150m

Appendix 4

7-cross

Configuration label m150l

Appendix 4

7-cross

Configuration label m150k

Appendix 4

7-cross

Configuration label m150j

Appendix 4

7-cross

Configuration label m150i

Appendix 4

7-cross

Configuration label m150h

Appendix 4

7-cross

Configuration label m150g

Appendix 4

7-cross

Configuration label m150f

Appendix 4

7-cross

Configuration label m150e

Appendix 4

7-cross

Configuration label m150d

Appendix 4

7-cross

Configuration label m150c

Appendix 4

7-cross

Configuration label m150b

Appendix 4

7-cross

Configuration label m150a

Appendix 4

7-cross

Configuration label a48m150

Appendix 4

7-cross

Configuration label b48

Appendix 4

7-cross

Configuration label mb28

Appendix 4

7-cross

Configuration label b24

Appendix 4

7-cross

Configuration label m102u

Appendix 4

7-cross

Configuration label m102t

Appendix 4

7-cross

Configuration label m102s

Appendix 4

7-cross

Configuration label m102r

Appendix 4

7-cross

Configuration label m102q

Appendix 4

7-cross

Configuration label m102p

Appendix 4

7-cross

Configuration label m102o

Appendix 4

7-cross

Configuration label m102n

Appendix 4

7-cross

Configuration label m102m

Appendix 4

7-cross

Configuration label m102l

Appendix 4

7-cross

Configuration label m102k

Appendix 4

7-cross

Configuration label m102j

Appendix 4

7-cross

Configuration label m102i

Appendix 4

7-cross

Configuration label m102h

Appendix 4

7-cross

Configuration label m102g

Appendix 4

7-cross

Configuration label m102f

Appendix 4

7-cross

Configuration label m102e

Appendix 4

7-cross

Configuration label m102d

Appendix 4

7-cross

Configuration label m102c

Appendix 4

7-cross

Configuration label m102b

Appendix 4

7-cross

Configuration label m102a

Appendix 4

7-cross

Configuration label b46

Appendix 4

7-cross

Configuration label b16

Appendix 4

7-cross

Configuration label b14

Appendix 4

7-cross

Configuration label a24m102

Appendix 4

7-cross

Configuration label ma24102b

Appendix 4

7-cross

Configuration label ma24102a

Appendix 4

7-cross

Configuration label x66i

Appendix 4

7-cross

Configuration label x66h

Appendix 4

7-cross

Configuration label x66g

Appendix 4

7-cross

Configuration label x66f

Appendix 4

7-cross

Configuration label x66e

Appendix 4

7-cross

Configuration label x66d

Appendix 4

7-cross

Configuration label x66c

Appendix 4

7-cross

Configuration label x66b

Appendix 4

7-cross

Configuration label x66a

Appendix 4

7-cross

Configuration label a2466

Appendix 4

7-cross

Configuration label m54g

Appendix 4

7-cross

Configuration label m54f

Appendix 4

7-cross

Configuration label m54e

Appendix 4

7-cross

Configuration label m54d

Appendix 4

7-cross

Configuration label m54c

Appendix 4

7-cross

Configuration label m54b

Appendix 4

7-cross

Configuration label m54a

Appendix 4

7-cross

Configuration label x42ak

Appendix 4

7-cross

Configuration label x42aj

Appendix 4

7-cross

Configuration label x42ai

Appendix 4

7-cross

Configuration label x42ah

Appendix 4

7-cross

Configuration label x42ag

Appendix 4

7-cross

Configuration label x42af

Appendix 4

7-cross

Configuration label x42ae

Appendix 4

7-cross

Configuration label x42ad

Appendix 4

7-cross

Configuration label x42ac

Appendix 4

7-cross

Configuration label x42ab

Appendix 4

7-cross

Configuration label x42aa

Appendix 4

7-cross

Configuration label x42z

Appendix 4

7-cross

Configuration label x42y

Appendix 4

7-cross

Configuration label x42x

Appendix 4

7-cross

Configuration label x42w

Appendix 4

7-cross

Configuration label x42v

Appendix 4

7-cross

Configuration label x42u

Appendix 4

7-cross

Configuration label x42t

Appendix 4

7-cross

Configuration label x42s

Appendix 4

7-cross

Configuration label x42r

Appendix 4

7-cross

Configuration label x42q

Appendix 4

7-cross

Configuration label x42p

Appendix 4

7-cross

Configuration label x42o

Appendix 4

7-cross

Configuration label x42n

Appendix 4

7-cross

Configuration label x42m

Appendix 4

7-cross

Configuration label x42l

Appendix 4

7-cross

Configuration label x42k

Appendix 4

7-cross

Configuration label x42j

Appendix 4

7-cross

Configuration label x42i

Appendix 4

7-cross

Configuration label x42h

Appendix 4

7-cross

Configuration label x42g

Appendix 4

7-cross

Configuration label x42f

Appendix 4

7-cross

Configuration label x42e

Appendix 4

7-cross

Configuration label x42d

Appendix 4

7-cross

Configuration label x42c

Appendix 4

7-cross

Configuration label x42b

Appendix 4

7-cross

Configuration label x42a

Appendix 4

7-cross

Configuration label mb78

Appendix 4

7-cross

Configuration label mb26

Appendix 4

7-cross

Configuration label mc78

Appendix 4

7-cross

Configuration label mc67

Appendix 4

7-cross

Configuration label mc16

Appendix 4

7-cross

Configuration label md26

Appendix 4

7-cross

Configuration label b58

Appendix 4

7-cross

Configuration label b15

Appendix 4

7-cross

Configuration label b12

Appendix 4

7-cross

Configuration label d46

Appendix 4

7-cross

Configuration label d14

Appendix 4

7-cross

Configuration label d12

Appendix 4

7-cross

Configuration label m18ce

Appendix 4

7-cross

Configuration label m18cd

Appendix 4

7-cross

Configuration label m18cc

Appendix 4

7-cross

Configuration label m18cb

Appendix 4

7-cross

Configuration label m18ca

Appendix 4

7-cross

Configuration label m18bz

Appendix 4

7-cross

Configuration label m18by

Appendix 4

7-cross

Configuration label m18bx

Appendix 4

7-cross

Configuration label m18bw

Appendix 4

7-cross

Configuration label m18bv

Appendix 4

7-cross

Configuration label m18bu

Appendix 4

7-cross

Configuration label m18bt

Appendix 4

7-cross

Configuration label m18bs

Appendix 4

7-cross

Configuration label m18br

Appendix 4

7-cross

Configuration label m18bq

Appendix 4

7-cross

Configuration label m18bp

Appendix 4

7-cross

Configuration label m18bo

Appendix 4

7-cross

Configuration label m18bn

Appendix 4

7-cross

Configuration label m18bm

Appendix 4

7-cross

Configuration label m18bl

Appendix 4

7-cross

Configuration label m18bk

Appendix 4

7-cross

Configuration label m18bj

Appendix 4

7-cross

Configuration label m18bi

Appendix 4

7-cross

Configuration label m18bh

Appendix 4

7-cross

Configuration label m18bg

Appendix 4

7-cross

Configuration label m18bf

Appendix 4

7-cross

Configuration label m18be

Appendix 4

7-cross

Configuration label m18bd

Appendix 4

7-cross

Configuration label m18bc

Appendix 4

7-cross

Configuration label m18bb

Appendix 4

7-cross

Configuration label m18ba

Appendix 4

7-cross

Configuration label m18az

Appendix 4

7-cross

Configuration label m18ay

Appendix 4

7-cross

Configuration label m18ax

Appendix 4

7-cross

Configuration label m18aw

Appendix 4

7-cross

Configuration label m18av

Appendix 4

7-cross

Configuration label m18au

Appendix 4

7-cross

Configuration label m18at

Appendix 4

7-cross

Configuration label m18as

Appendix 4

7-cross

Configuration label m18ar

Appendix 4

7-cross

Configuration label m18aq

Appendix 4

7-cross

Configuration label m18ap

Appendix 4

7-cross

Configuration label m18ao

Appendix 4

7-cross

Configuration label m18an

Appendix 4

7-cross

Configuration label m18am

Appendix 4

7-cross

Configuration label m18al

Appendix 4

7-cross

Configuration label m18ak

Appendix 4

7-cross

Configuration label m18aj

Appendix 4

7-cross

Configuration label m18ai

Appendix 4

7-cross

Configuration label m18ah

Appendix 4

7-cross

Configuration label m18ag

Appendix 4

7-cross

Configuration label m18af

Appendix 4

7-cross

Configuration label m18ae

Appendix 4

7-cross

Configuration label m18ad

Appendix 4

7-cross

Configuration label m18ac

Appendix 4

7-cross

Configuration label m18ab

Appendix 4

7-cross

Configuration label m18aa

Appendix 4

7-cross

Configuration label m18z

Appendix 4

7-cross

Configuration label m18y

Appendix 4

7-cross

Configuration label m18x

Appendix 4

7-cross

Configuration label m18w

Appendix 4

7-cross

Configuration label m18v

Appendix 4

7-cross

Configuration label m18u

Appendix 4

7-cross

Configuration label m18t

Appendix 4

7-cross

Configuration label m18s

Appendix 4

7-cross

Configuration label m18r

Appendix 4

7-cross

Configuration label m18q

Appendix 4

7-cross

Configuration label f67

Appendix 4

7-cross

Configuration label f57

Appendix 4

7-cross

Configuration label f56

Appendix 4

7-cross

Configuration label m18p

Appendix 4

7-cross

Configuration label m18o

Appendix 4

7-cross

Configuration label m18n

Appendix 4

7-cross

Configuration label m18m

Appendix 4

7-cross

Configuration label m18l

Appendix 4

7-cross

Configuration label m18k

Appendix 4

7-cross

Configuration label m18j

Appendix 4

7-cross

Configuration label m18i

Appendix 4

7-cross

Configuration label m18h

Appendix 4

7-cross

Configuration label m18g

Appendix 4

7-cross

Configuration label m18f

Appendix 4

7-cross

Configuration label m18e

Appendix 4

7-cross

Configuration label m18d

Appendix 4

7-cross

Configuration label m18c

Appendix 4

7-cross

Configuration label m18b

Appendix 4

7-cross

Configuration label m18a

Appendix 4

7-cross

Configuration label me29

Appendix 4

7-cross

Configuration label me28

Appendix 4

7-cross

Configuration label e48

Appendix 4

7-cross

Configuration label md24

Appendix 4

7-cross

Configuration label md16

Appendix 4

7-cross

Configuration label d28

Appendix 4

7-cross

Configuration label d18

Appendix 4

7-cross

Configuration label mc13

Appendix 4

7-cross

Configuration label c48

Appendix 4

7-cross

Configuration label c46

Appendix 4

7-cross

Configuration label c27

Appendix 4

7-cross

Configuration label c24

Appendix 4

7-cross

Configuration label c14

Appendix 4

7-cross

Configuration label mb68

Appendix 4

7-cross

Configuration label mb57

Appendix 4

7-cross

Configuration label mb56

Appendix 4

7-cross

Configuration label b45

Appendix 4

7-cross

Configuration label b27

Appendix 4

7-cross

Configuration label ma49

Appendix 4

7-cross

Configuration label ma29

Appendix 4

7-cross

Configuration label ma28

Appendix 4

7-cross

Configuration label a89

Appendix 4

7-cross

Configuration label a48

Appendix 4

7-cross

Configuration label a24

Appendix 4

7-cross

Configuration label m10ak

Appendix 4

7-cross

Configuration label m10aj

Appendix 4

7-cross

Configuration label m10ai

Appendix 4

7-cross

Configuration label m10ah

Appendix 4

7-cross

Configuration label m10ag

Appendix 4

7-cross

Configuration label m10af

Appendix 4

7-cross

Configuration label m10ae

Appendix 4

7-cross

Configuration label m10ad

Appendix 4

7-cross

Configuration label m10ac

Appendix 4

7-cross

Configuration label m10ab

Appendix 4

7-cross

Configuration label m10aa

Appendix 4

7-cross

Configuration label m10z

Appendix 4

7-cross

Configuration label m10y

Appendix 4

7-cross

Configuration label m10x

Appendix 4

7-cross

Configuration label m10w

Appendix 4

7-cross

Configuration label m10v

Appendix 4

7-cross

Configuration label m10u

Appendix 4

7-cross

Configuration label m10t

Appendix 4

7-cross

Configuration label m10s

Appendix 4

7-cross

Configuration label m10r

Appendix 4

7-cross

Configuration label m10q

Appendix 4

7-cross

Configuration label m10p

Appendix 4

7-cross

Configuration label m10o

Appendix 4

7-cross

Configuration label m10n

Appendix 4

7-cross

Configuration label m10m

Appendix 4

7-cross

Configuration label m10l

Appendix 4

7-cross

Configuration label m10k

Appendix 4

7-cross

Configuration label m10j

Appendix 4

7-cross

Configuration label f29

Appendix 4

7-cross

Configuration label m10i

Appendix 4

7-cross

Configuration label m10h

Appendix 4

7-cross

Configuration label m10g

Appendix 4

7-cross

Configuration label m10f

Appendix 4

7-cross

Configuration label m10e

Appendix 4

7-cross

Configuration label m10d

Appendix 4

7-cross

Configuration label m10c

Appendix 4

7-cross

Configuration label m10b

Appendix 4

7-cross

Configuration label m10a

Appendix 4

7-cross

Configuration label me69

Appendix 4

7-cross

Configuration label e46

Appendix 4

7-cross

Configuration label me39

Appendix 4

7-cross

Configuration label me36

Appendix 4

7-cross

Configuration label me26

Appendix 4

7-cross

Configuration label e16

Appendix 4

7-cross

Configuration label e14

Appendix 4

7-cross

Configuration label md67

Appendix 4

7-cross

Configuration label md27

Appendix 4

7-cross

Configuration label mc18

Appendix 4

7-cross

Configuration label c29

Appendix 4

7-cross

Configuration label c12

Appendix 4

7-cross

Configuration label ma69

Appendix 4

7-cross

Configuration label ma67

Appendix 4

7-cross

Configuration label ma39

Appendix 4

7-cross

Configuration label ma36

Appendix 4

7-cross

Configuration label ma27

Appendix 4

7-cross

Configuration label ma26

Appendix 4

7-cross

Configuration label a58

Appendix 4

7-cross

Configuration label a56

Appendix 4

7-cross

Configuration label a46

Appendix 4

7-cross

Configuration label a16

Appendix 4

7-cross

Configuration label a14

Appendix 4

7-cross

Configuration label m2az

Appendix 4

7-cross

Configuration label m2ay

Appendix 4

7-cross

Configuration label m2ax

Appendix 4

7-cross

Configuration label m2aw

Appendix 4

7-cross

Configuration label m2av

Appendix 4

7-cross

Configuration label m2au

Appendix 4

7-cross

Configuration label m2at

Appendix 4

7-cross

Configuration label m2as

Appendix 4

7-cross

Configuration label m2ar

Appendix 4

7-cross

Configuration label m2aq

Appendix 4

7-cross

Configuration label m2ap

Appendix 4

7-cross

Configuration label m2ao

Appendix 4

7-cross

Configuration label m2an

Appendix 4

7-cross

Configuration label m2am

Appendix 4

7-cross

Configuration label m2al

Appendix 4

7-cross

Configuration label m2ak

Appendix 4

7-cross

Configuration label m2aj

Appendix 4

7-cross

Configuration label m2ai

Appendix 4

7-cross

Configuration label m2ah

Appendix 4

7-cross

Configuration label m2ag

Appendix 4

7-cross

Configuration label m2af

Appendix 4

7-cross

Configuration label m2ae

Appendix 4

7-cross

Configuration label m2ad

Appendix 4

7-cross

Configuration label m2ac

Appendix 4

7-cross

Configuration label m2ab

Appendix 4

7-cross

Configuration label m2aa

Appendix 4

7-cross

Configuration label m2z

Appendix 4

7-cross

Configuration label m2y

Appendix 4

7-cross

Configuration label m2x

Appendix 4

7-cross

Configuration label m2w

Appendix 4

7-cross

Configuration label m2v

Appendix 4

7-cross

Configuration label m2u

Appendix 4

7-cross

Configuration label m2t

Appendix 4

7-cross

Configuration label m2s

Appendix 4

7-cross

Configuration label m2r

Appendix 4

7-cross

Configuration label m2q

Appendix 4

7-cross

Configuration label m2p

Appendix 4

7-cross

Configuration label f28

Appendix 4

7-cross

Configuration label f26

Appendix 4

7-cross

Configuration label f25

Appendix 4

7-cross

Configuration label m2o

Appendix 4

7-cross

Configuration label m2n

Appendix 4

7-cross

Configuration label m2m

Appendix 4

7-cross

Configuration label m2l

Appendix 4

7-cross

Configuration label m2k

Appendix 4

7-cross

Configuration label m2j

Appendix 4

7-cross

Configuration label m2i

Appendix 4

7-cross

Configuration label m2h

Appendix 4

7-cross

Configuration label m2g

Appendix 4

7-cross

Configuration label m2f

Appendix 4

7-cross

Configuration label m2e

Appendix 4

7-cross

Configuration label m2d

Appendix 4

7-cross

Configuration label m2c

Appendix 4

7-cross

Configuration label m2b

Appendix 4

7-cross

Configuration label m2a

Appendix 4

7-cross

Configuration label me38

Appendix 4

7-cross

Configuration label e19

Appendix 4

7-cross

Configuration label me18

Appendix 4

7-cross

Configuration label me17

Appendix 4

7-cross

Configuration label e13

Appendix 4

7-cross

Configuration label md68

Appendix 4

7-cross

Configuration label c69

Appendix 4

7-cross

Configuration label c68

Appendix 4

7-cross

Configuration label mc26

Appendix 4

7-cross

Configuration label mc19

Appendix 4

7-cross

Configuration label c17

Appendix 4

7-cross

Configuration label b18

Appendix 4

7-cross

Configuration label b17

Appendix 4

7-cross

Configuration label a17m2

Appendix 4

7-cross

Configuration label ma47

Appendix 4

7-cross

Configuration label ma45

Appendix 4

7-cross

Configuration label ma38

Appendix 4

7-cross

Configuration label ma35

Appendix 4

7-cross

Configuration label ma18

Appendix 4

7-cross

Configuration label ma17

Appendix 4

7-cross

Configuration label a57

Appendix 4

7-cross

Configuration label a25

Appendix 4

7-cross

Configuration label a23

Appendix 4

7-cross

Configuration label a19

Appendix 4

7-cross

Configuration label a13

Appendix 4

7-cross

Configuration label ma37

Appendix 4

7-cross

Configuration label ma34

Appendix 4

7-cross

Configuration label a59

Appendix 4

7-cross

Configuration label a15

Appendix 4

7-cross

Configuration label a12

Appendix 5
